# The Effect of Clinical Sandblasting With Different Powders on the Surface Roughness of Cores for Metal-Ceramic Crowns and Their Fracture Resistance After the Addition of Repair Material: An In-Vitro Study

**DOI:** 10.7759/cureus.33012

**Published:** 2022-12-27

**Authors:** Mohammed Yassin, Shatha Salih

**Affiliations:** 1 Department of Conservative Dentistry and Endodontics, Hawler Medical University, College of Dentistry, Erbil, IRQ

**Keywords:** intraoral repair, metal-ceramic crowns, surface roughness, silica, sandblasting

## Abstract

Background

One of the most frequently encountered issues with metal-ceramic restorations is the fracture of veneering porcelain. This in-vitro study aims to evaluate the effect of clinical sandblasting with 50 μm aluminum oxide and 30 μm silica-coated particles on the surface roughness of metal cores and the subsequent effect on their fracture resistance after the addition of specific adhesive and packable composite as a repair material.

Methodology

Metal cores (n = 21) were digitally designed and three-dimensionally printed by selective laser melting (SLM) technique. These cores were randomly divided into three groups. Group A (n = 8) was sandblasted with 50 μm aluminum oxide and veneered with light cure composite. Group B (n = 8) was sandblasted with 30 μm silica-coated particles and veneered with light cure composite. Group C control group (n = 5) was sandblasted in the laboratory with 250 μm aluminum oxide and veneered with porcelain. All specimens were tested for surface roughness by a stylus profilometer. After adding the veneering material, all specimens were subjected to a fracture resistance test through a universal testing machine.

Results

One-way analysis of variance test showed a significantly higher difference for the specimens sandblasted in the laboratory using 250 μm aluminum oxide. Fracture resistance values showed no significant difference between groups A and B.

Conclusions

Groups A and B showed no significant difference in surface roughness, but their fracture resistance values were above the acceptable clinical limit. Despite the rough nature of metal cores fabricated by the SLM technique, sandblasting with silica-coated particles may be an effective way to optimize the fracture resistance of the repair material because it provides the basis for chemical adhesion.

## Introduction

Dental esthetics can be defined as the science and art of enhancing the aesthetics and function of the teeth, oral cavity, and facial symmetry via the application of certain knowledge and methods [1]. In the late 1700s, ceramics were introduced as restorative materials in dentistry, taking advantage of their ability to mimic the form and color of natural teeth [2]. The three basic types of materials that are used for indirect dental restorations include metal alloys (both all-metal and metal-ceramic), ceramics, and resin-based composites [3]. Porcelain fused to metal (PFM) restorations have the benefit of combining clinical durability with acceptable cosmetic results. However, there are post-insertion complications with metal-ceramic crowns and fixed partial dentures [4]. Biological or technical complications are also possible. One of the most common issues with metal-ceramic restorations is the chipping of the veneering material [5]. It may not be the most practical alternative to replace the entire restoration because it is time-consuming, expensive, and there is a risk of traumatizing the abutment [6]. On the other hand, repairing the broken porcelain intraorally is simple and offers a cost and time-effective alternative to the patient and the dentist, ensuring that both function and esthetics are restored [7].

Laser melting via computer-aided design/computer-aided manufacture (CAD/CAM) is an additional production technology for metal copings of metal-ceramic crowns [8]. The constraints of conventional waxing and the lost-wax technique do not apply to this method. Selective laser melting (SLM) uses high-temperature laser beams to selectively heat the metal framework, which is in a powder form, and employs CAD data generated from the framework’s design for this purpose [9].

Sandblasting, grinding, acid or heat etching, or combinations thereof are some of the most frequent surface roughening techniques [10,11]. One of the most popular surface treatment methods, airborne-particle abrasion (sandblasting), causes micro undercuts, increases the substrate’s surface roughness, and enhances micro-mechanical bonding agent retention [12]. There are a variety of particle sizes (ranging from 25 μm to 250 μm) and compositions (such as ordinary alumina or silica-coated particles) available for abrasion by airborne particles [13,14]. In the existing literature, there are little data regarding the effect of clinical sandblasting on metal cores fabricated by the SLM technique; hence, this study aims to investigate the effect of clinical sandblasting with 50 μm aluminum oxide and 30 μm silica-coated particles on the surface roughness of metal cores and the subsequent effect on their fracture resistance after the addition of repair material.

## Materials and methods

Abutment preparation

**Figure 1:**
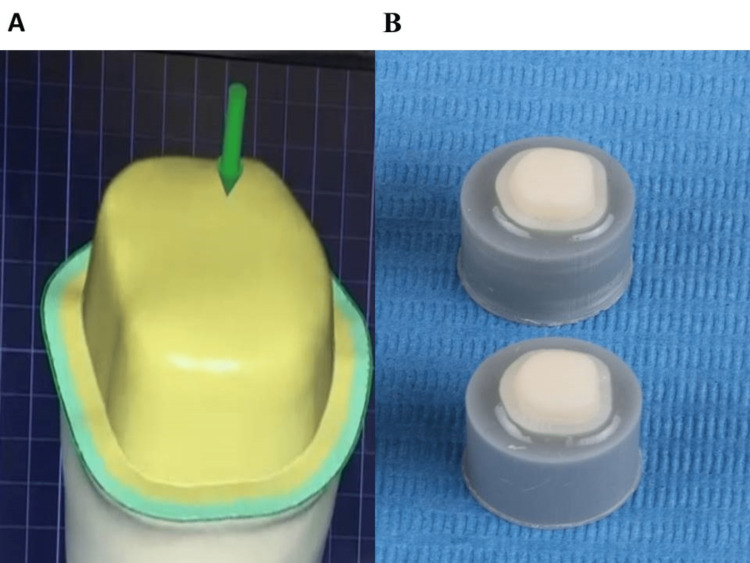
A: Digital design of the prepared abutment B: Three-dimensional-printed polymethyl methacrylate definitive dies.

The lower right first molar was digitally designed using Exocad software (Exocad 3.0 Galway) according to the dimensions of a natural tooth, as documented by Chatterjee [15]. No scan of a natural tooth was performed. Using the same software, a digital preparation was performed with the following guidelines: occlusal reduction - 1.5 mm at the center and 2 mm at the cusp tip with 45-degree beveling. The other surfaces were reduced with a taper of 6 degrees. Finishing line: 1.3 mm all around the shoulder with internal roundation (Figure 1A). A total of 21 definitive polymethyl methacrylate (PMMA) (DETAX, Germany) casts were digitally printed using Arum 5x-300 Pro milling machine (Arum Dentistry) corresponding to the previously mentioned parameters. Each cast was attached to a three-dimensional (3D)-printed, cylindrical acrylic block (Mazic, Vericom Co., Ltd., Gangwon-do, Korea) with a height of 20 mm and a diameter of 16 mm (Figure 1B). These are considered the abutments to receive the cores and the crowns.

Fabrication of metal cores and metal-ceramic crowns

**Figure 2:**
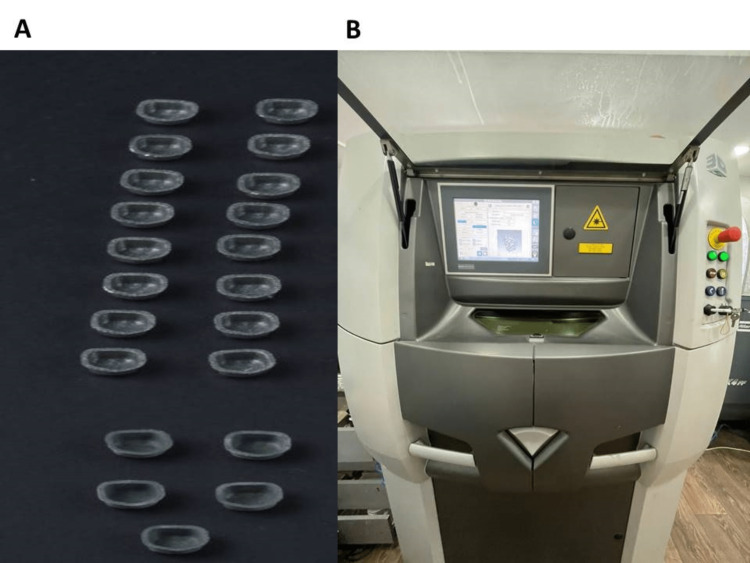
A: Metal cores. B: ProX 100 three-dimensional printer.

Using Exocad 3.0 Galway software, metal cores (n = 21) were designed with a thickness of 0.5 mm (Figure 2A) according to manufacturer instructions and 3D printed by the SLM technique with cobalt-chrome metal powder (3D Systems, Inc.) by ProX 100 3D printer (3D Systems, Inc., Rock Hill, SC, USA) (Figure 2B) with an accuracy of 30 μ per each slice, followed by heating up to 800°C for one hour using a program at p310 furnace (Ivoclar Vivadent AG, Schaan, Liechtenstein). These cores were randomly divided into the following three groups: Group A (n = 8) (sandblasted clinically with 50 μm aluminum oxide), group B (n = 8) (sandblasted clinically with 30 μm silica-coated particles), and group C (n = 5) (control group: sandblasted in the laboratory with 250 μm aluminum oxide). Specimens of the control group were veneered with porcelain according to manufacturer instructions (Vintage Pro, SHOFU Dental GmbH, Ratingen, Germany).

Sandblasting

**Figure 3:**
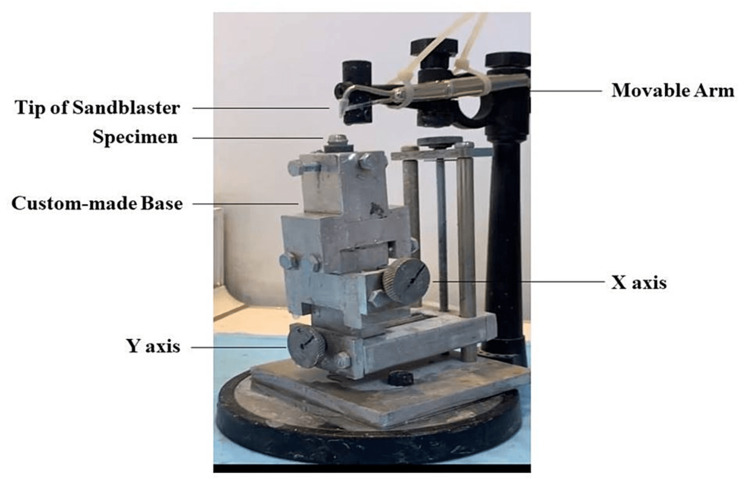
Sandblasting technique: each complete Y and X knob rotation moves the base 1 mm on its axis.

Specimens of the control group were sandblasted in the laboratory using 250 μm aluminum oxide (Korox; BEGO Medical) at 2 bar pressure from a 50 mm distance as a setting for laboratory work. The specimens from group A were clinically sandblasted with 50 μm aluminum oxide (Dentify GmbH, Germany), while the specimens from group B were clinically sandblasted with 30 μm silica-coated particles (3M™ Cojet™ Sand). Clinical sandblasting parameters were set as follows: 2.5 bar pressure from a 10 mm distance for 10 seconds using a clinical sandblasting device (AquaCare, Velopex International, London, UK). The sandblaster handle was attached to a customized dental surveyor (Ney Surveyor, Ney Dental, Bloomfield, CT, USA) to allow for standard movement during the process of sandblasting (Figure 3).

Surface roughness test

**Figure 4:**
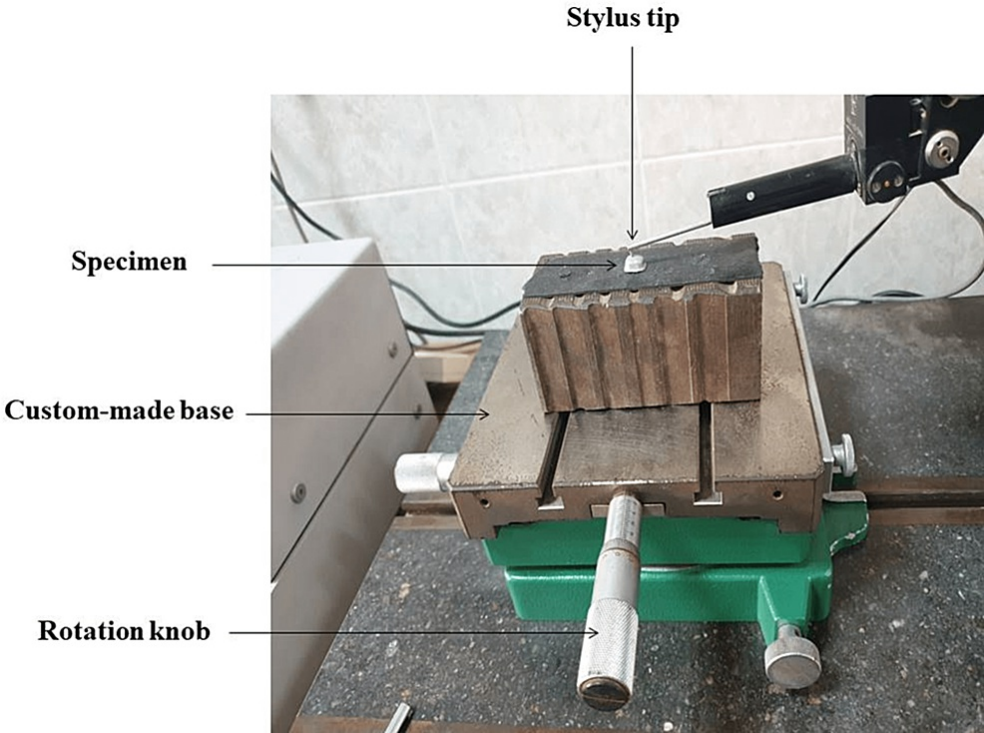
Surface roughness test.

All samples were tested for surface roughness using the Taylor-Hobson profilometer (Figure 4). Specimens of the control group were tested twice, before and after sandblasting. Three readings for each specimen were recorded at a 1 mm distance between each line, one in the center and the other two at a 1 mm distance above and below, and the mean value was calculated.

Cementation

All specimens were cemented using dual-cure self-adhesive resin cement, TheraCem (Bisco, Schaumburg, USA), following manufacturer instructions. A 5 kg weight was used to keep the samples in place during the primary cement setting to ensure uniform seating pressure.

Application of repair material

**Figure 5:**
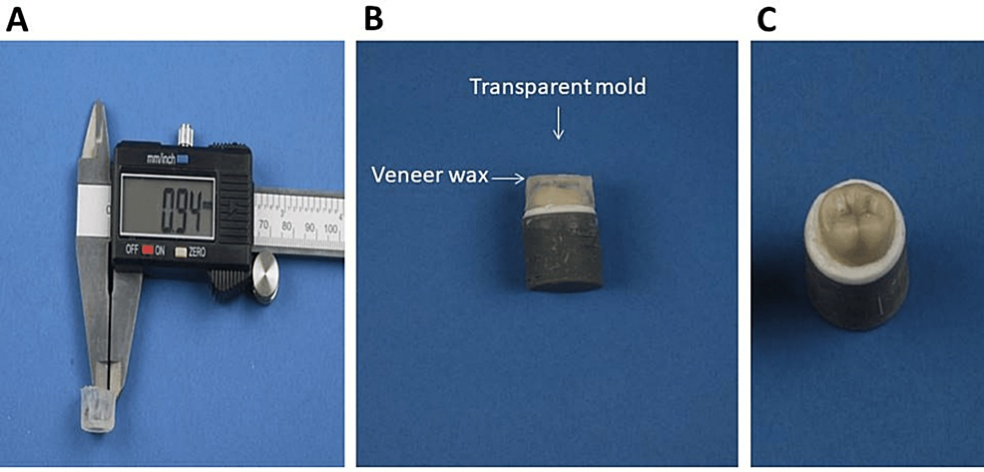
A: Thickness of the mold. B: Adaptation of the mold. C: Addition of the repair material.

The composite build-up was performed on all specimens from groups A and B using light-cure resin composite (3M Filtek Z350 XT). A transparent mold with a thickness of approximately 1 mm (Figure 5A) was fabricated using clear polyvinyl siloxane (EXACLEAR; GC Corp.) on a randomly chosen specimen from the control group to control the thickness of the composite material. A layer of veneer wax (Renfert GmbH, Hilzingen, Germany) was added beneath the finishing line of the definitive die to block the undercut and control the fit of the mold (Figure 5B). Before the addition of the veneering composite, the porcelain repair kit (Intraoral Repair Kit, Bisco Inc., Schaumburg, IL) was used according to the manufacturer’s instructions. One coat of Z-Prime Plus was applied and dried with an air syringe for three to five seconds. A thin layer of porcelain bonding resin was applied and spread evenly on the surface, and then air-thinned for three to five seconds. For each specimen, the mold was loaded with two capsules (3M Filtek Z350 XT) and secured over the specimen. The excess composite was removed with a micro brush and then light-cured for 20 seconds for each occlusal, buccal, lingual, mesial, and distal surface using a light-curing pen (Eighteenth, Changzhou, China) at an intensity of 1,000 mW/cm² from a distance of 1-2 mm. After the removal of the transparent mold (Figure 5C), each surface was light-cured for another 20 seconds. The specimens were then kept in 37°C distilled water for one week. All specimens were then subjected to 1,000 rounds of thermal cycling between 5 ± 2°C and 55 ± 2°C for 30 seconds in each bath with five-second intervals between the baths.

Fracture load test

**Figure 6:**
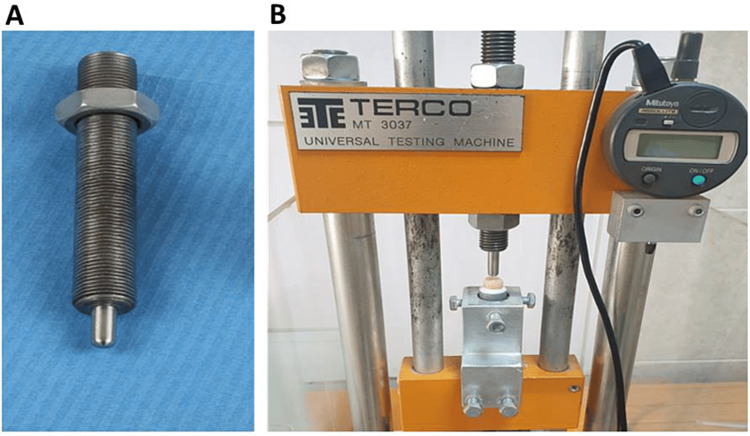
A: Custom-made indenter B: Position of the specimen.

All specimens were then loaded by a universal testing machine (TERCO MT 3037 Terco I&S AB, Sweden). Each specimen was fixed in a custom-made metallic base, and the pressure was delivered via a vertically moving rod with a semi-spherical head of 6 mm in diameter (Figure 6A), with a cross speed of 1 mm/minute. The loading piston was positioned at the center of the occlusal surface (Figure 6B). Three examiners examined the position of the specimen to ensure its accuracy.

Statistical analysis

Following a one-way analysis of variance (ANOVA), the normality and homogeneity of variance were tested using Shapiro-Wilk and Levene’s tests, respectively. Paired t-tests were computed for groups measured twice, and independent two-sample t-tests were used to compare the two groups. The Bonferroni test was used to detect multiple comparisons among the experimental groups mentioned above. SPSS version 25 (IBM Corp., Armonk, NY, USA) was used to run all statistical tests. A significant difference was set at p < 0.05.

## Results

Table 1 shows the descriptive statistics of surface roughness measurements where the data are presented as mean ± SD. No statistically significant difference was found between groups C before sandblasting, A, and B.

**Table 1:** Statistical paired t-test and one-way ANOVA test for surface roughness values (μm). SA = sandblasting; ANOVA = analysis of variance

Statistical test	Group	N	Mean ± SD	Minimum	Maximum	Test value (p-value)
Paired test	C - Before SA	5	1.553 ± 0.112	1.420	1.680	-11.018 (<0.001)
	C - After SA	5	2.280 ± 0.100	2.12	2.35	
One-way ANOVA	C - Before SA	5	1.553 ± 0.112	1.420	1.680	0.151 (0.861)
	A	8	1.588 ± 0.203	1.400	1.900	
	B	8	1.535 ± 0.215	1.200	1.833	

Table 2 shows the descriptive statistics of fracture resistance measurements where the data are presented as mean ± SD.

**Table 2:** Descriptive statistical result for fracture resistance measure per group (N).

Group	N	Mean ± SD	Standard error	Minimum	Maximum
C	5	2,310.000 ± 527.210	235.775	1,730.000	2,910.000
A	8	1,311.250 ± 301.777	106.694	930.000	1,770.000
B	8	1,328.750 ± 299.306	105.821	1,110.000	2,000.000

Although group B showed higher values of fracture resistance than group A, no statistically significant result was noted, with a p-value of 1 (Table 3).

**Table 3:** Bonferroni pairwise comparison test result. PFM = porcelain fused to metal

Group		Mean difference (I-J)	Standard error	Significance	95% confidence interval	
					Lower bound	Upper bound
C (PFM)	A	998.750^*^	207.139	<0.001	452.079	1,545.421
	B	981.250^*^	207.139	<0.001	434.579	1,527.921
A	B	-17.500	181.673	1.000	-496.962	461.962

**Figure 7:**
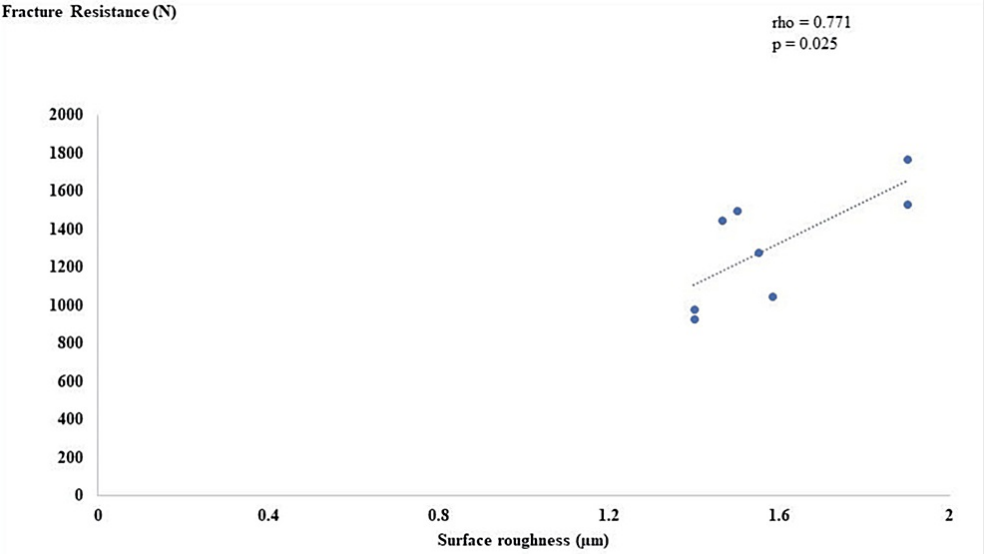
Correlation between surface roughness and fracture resistance in group A.

**Figure 8:**
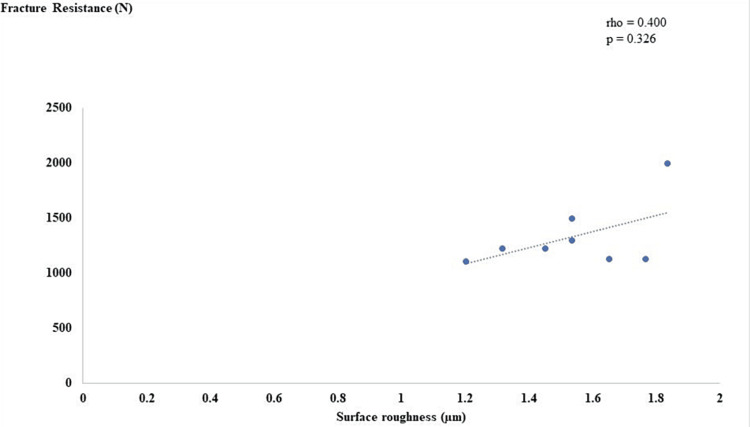
Correlation between surface roughness and fracture resistance in group B.

Linear regression modeling was done to highlight the variability between surface roughness and fracture resistance. The scatter plot displayed that there was a positive linear relationship between surface roughness and fracture resistance, as shown in Figure 7 and Figure 8.

## Discussion

The gold standard in posterior tooth indirect restorations is a metal-ceramic fixed partial prosthesis [16]. When it comes to metal-ceramic restorations, the chipping of the veneering ceramic is considered to be one of the most common issues that can arise [17]. In this study, preparation parameters were selected to provide sufficient thickness for both metal cores and veneering material according to manufacturer instructions. Because the modulus of elasticity of PMMA is comparable to that of human dentin [18], it was decided that PMMA would be the material of choice for the production of definitive dies.

The SLM technique can create Co-Cr restorations with equivalent or superior quality to casting and CAD/CAM machining in a fraction of the time and expense [19]. In addition to producing restorations with more uniform quality, the SLM technique standardizes the process of shaping restorations, uses fewer personnel, and has the potential to produce greater accuracy because it eliminates multiple procedural steps such as waxing up, flasking, and casting [20]. It is worth mentioning that the surface roughness of Co-Cr specimens is affected by manufacturing processes. After additive manufacturing of Co-Cr specimens, a post-processing heat treatment of Co-Cr metal alloys is necessary to alleviate internal tensions induced by temperature gradients during the process. The surface roughness of SLM specimens is determined by their layer-by-layer configuration [21].

An airborne-particle abrasion method (most typically sandblasting using alumina particles) is frequently used to remove contaminants, roughen the substrate surface, and alter the wettability and energy of the substrate [22]. In this study, the sandblasting parameters, including the size of particles, distance, pressure, and duration, were selected based on the previous study performed by Okada et al. as this combination showed the most favorable outcomes regarding flexural strength [14]. Regarding sandblasting effect, the results of this study revealed that there was no significant change in the surface roughness of the metal cores after clinical sandblasting with either 50 μm aluminum oxide or 30 μm silica-coated particles. This could be explained by the rough nature of the metal surface fabricated by the SLM technique, which is supported by the findings of Alqahtan et al. as there were increased porosities and micro-irregularities evident on the untreated metal surface [23]. The results of this study are also consistent with the findings of Revilla-León et al. as it showed that the surface roughness readings of Co-Cr specimens produced by the milling technique (subtractive) which were sandblasted using 100 μm aluminum oxide were significantly lower than untreated specimens produced by the SLM technique [24]. Sandblasting with silica-coated particles showed the least roughness value. This could be attributed to the chemical interaction between the silica particles and the metal substrate, which maintains the silica particles lodged in the metal substrate’s surface; thus, filling the micro-porosities [25]. Laboratory sandblasting with 250 μm aluminum oxide showed significantly higher roughness values compared to the other groups, which could be attributed to the larger particle size of aluminum oxide powder [26].

The metal cores for both groups A and B were treated with a specific repairing kit which provides the basis for chemical bonding with the metal oxide generated on base metal alloys. This is because it contains 10-methacryloyloxydecyl dihydrogen phosphate (MDP), which may increase the binding strength to base metal alloys, as stated by Sadighpour et al. [27]. A vinyl group on the other end of the molecule aids polymerization with the resin matrix’s unsaturated carbon bonds. An ester chain with 10 carbons serves as a spacer between these two active groups [28].

Regarding fracture resistance, the results of this study showed significantly higher values for the control group, which could be explained by the difference in the processing technique and veneering material [29]. Groups A and B showed no significant differences with slightly higher values in groups B, which could be attributed to the fact that sandblasting with silica coating embeds silica particles in the surface of the substrate, producing a physically and chemically active outer surface layer (oxide layer), which promotes a chemical adhesion with the phosphate monomer (MDP), resulting in a stronger bonding [30]. It is worth mentioning that all specimens exhibited higher values than the average bite force in the first molar region, as documented by Khan et al. [31].

## Conclusions

Clinical sandblasting of metal cores fabricated by the SLM technique with either 50 μm aluminum oxide or 30 μm silica-coated particles did not significantly affect the surface roughness, unlike the laboratory sandblasting with 250 μm aluminum oxide which significantly increased the surface roughness. Regarding fracture resistance, all specimens showed values above the acceptable clinical limit.

## Figures and Tables

**Figure 9:**
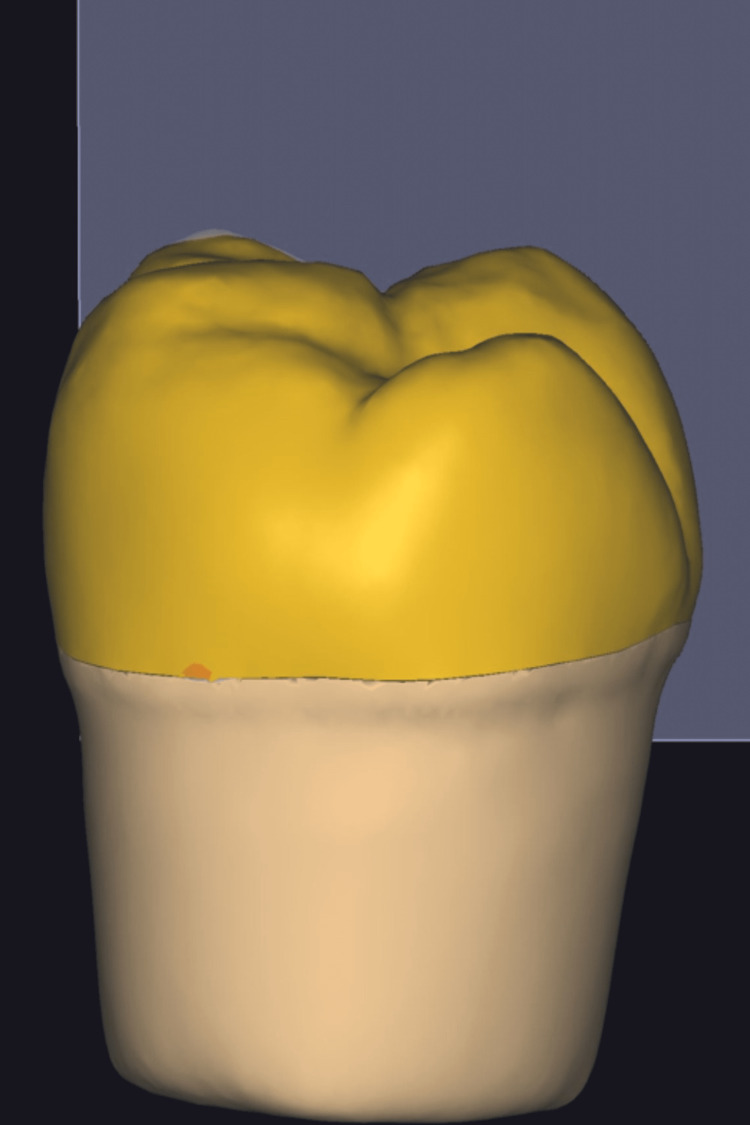
Digital design of natural tooth.

**Figure 10:**
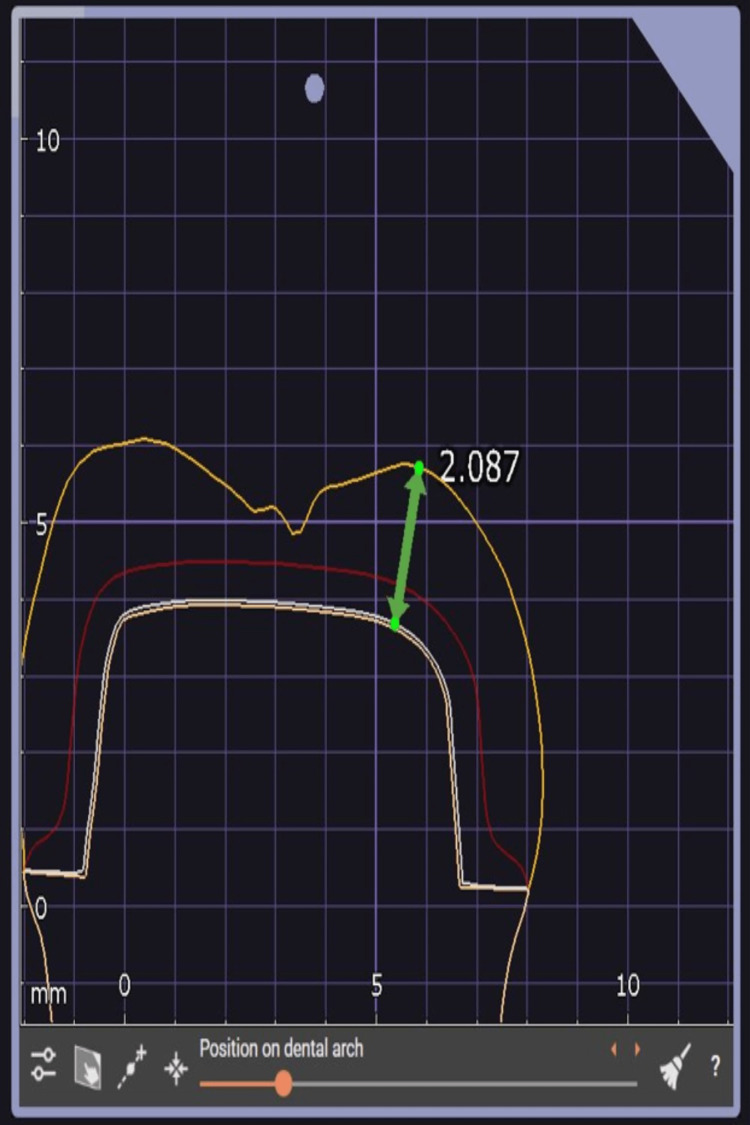
Cusp tip reduction.

**Figure 11:**
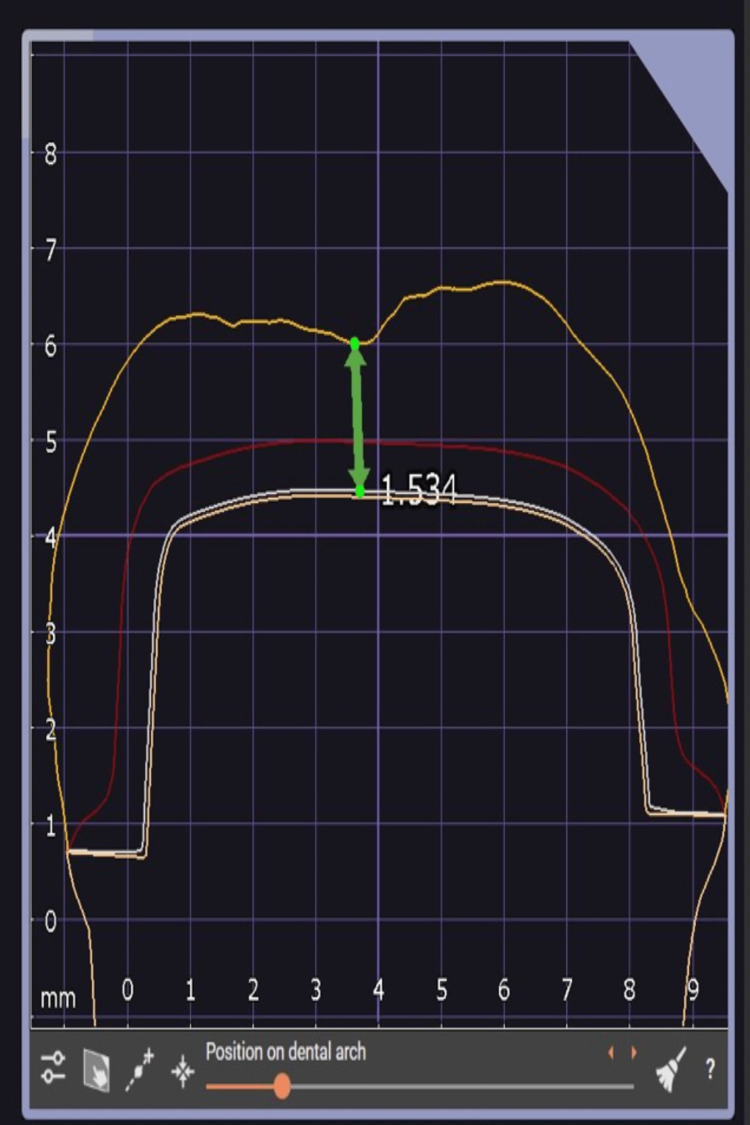
Cusp reduction at the center.

**Figure 12:**
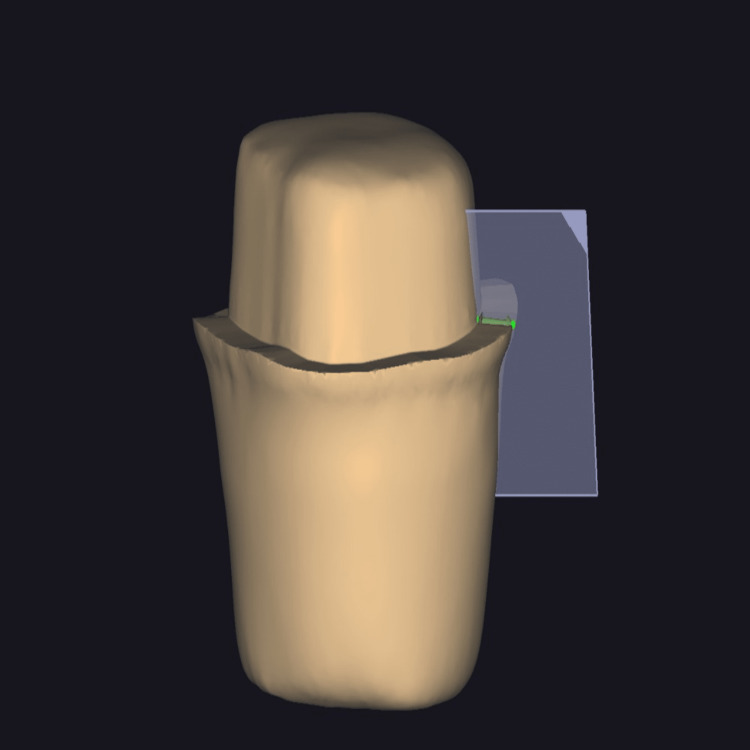
Final preparation design.

## Appendices

**Table 4:** Raw data.

Group	Surface roughness before sandblasting/μm	Mean/μm	Surface roughness after sandblasting/μm	Mean/μm	Fracture resistance (N)	Mean (N)
C	1.7/1.85/1.5		2.0/2.55/2.2		1,790	
	1.2/2.4/1.0		2.3/2.5/2.25		2,460	
	1.45/1.7/1.3	1.553	1.9/2.35/2.1	2.28	1,730	2,310
	1.5/1.8/1.65		2.2/2.3/2.5		2,660	
	1.3/1.55/1.4		2.1/2.55/2.4		2,910	
A			1.5/1.5/1.65		1,280	
			1.7/1.3/1.75		1,050	
			1.3/1.5/1.4		980	
			1.9/1.7/2.1	1.5875	1,770	
			1.6/1.4/1.5		1,500	1,311.25
			1.4/1.7/1.3		1,450	
			1.4/1.35/1.45		930	
			2.1/1.7/1.9		1,530	
B			1.4/0.9/1.3		1,110	
			1.7/1.4/1.5		1,300	
			1.3/1.3/1.35		1,230	
			1.7/1.5/1.4	1.5354	1,500	
			1.7/2.0/1.6		1,130	1,328.75
			1.45/1.5/1.4		1,230	
			2.0/1.5/1.45		1,130	
			1.6/2.0/1.9		2,000	
